# Plasminogen activator Inhibitor-1 in systemic sclerosis: A nexus of fibrosis, vasculopathy, and senescence

**DOI:** 10.1016/j.bj.2025.100906

**Published:** 2025-08-15

**Authors:** Takuya Takahashi, Takehiro Takahashi, Yoshihide Asano

**Affiliations:** Department of Dermatology, Tohoku University Graduate School of Medicine, Sendai, Japan

**Keywords:** Plasminogen activator inhibitor-1, Systemic sclerosis, Fibrosis, Vasculopathy, Cellular senescence

## Abstract

Plasminogen activator inhibitor-1 (PAI-1), a key regulator of the fibrinolytic system, has emerged as a multifaceted contributor to the pathogenesis of systemic sclerosis (SSc). Beyond its classical role in inhibiting plasminogen activation, PAI-1 is implicated in the dysregulation of vascular remodeling, promotion of fibrosis, modulation of immune responses, and the maintenance of cellular senescence—all of which are hallmarks of SSc. Notably, elevated PAI-1 expression has been observed in both patient-derived tissues and experimental models of the disease. Mice deficient in the urokinase-type plasminogen activator receptor (uPAR), which functions with its ligand urokinase-type plasminogen activator (uPA) in the plasminogen activation system, exhibit impaired fibrinolysis and spontaneously develop vasculopathy and fibrosis, closely mirroring human SSc. These findings underscore the pathogenic relevance of the uPA–uPAR–PAI-1 axis in disease progression. Moreover, recent studies suggest that pharmacological inhibition of PAI-1 may not only ameliorate fibrosis and vascular abnormalities but also promote the clearance of senescent cells, thereby interrupting the vicious cycle of chronic inflammation and maladaptive tissue remodeling in SSc. This review highlights the emerging roles of PAI-1 in SSc pathophysiology and explores its potential as a novel therapeutic target for disease modification.

## 1 Introduction

**Fig. 1:**
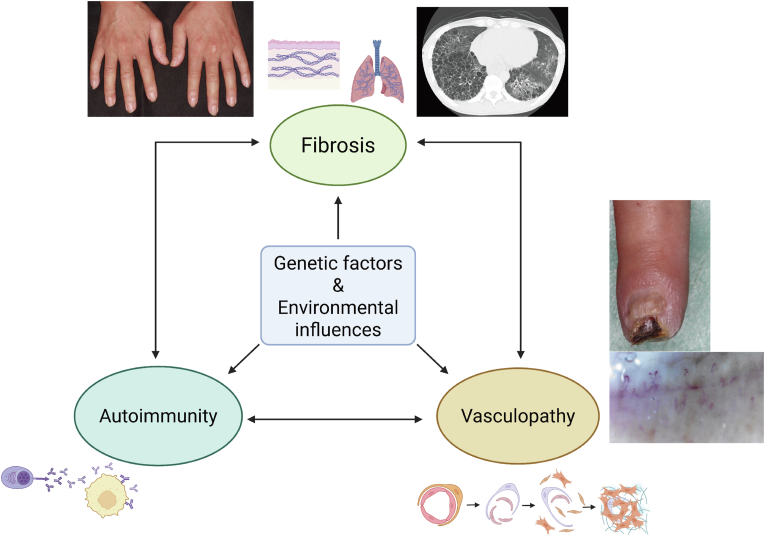
Overview of the pathogenesis of systemic sclerosis (SSc). A schematic illustration depicting the major pathological hallmarks of SSc, including immune dysregulation, vascular dysfunction, and progressive tissue fibrosis.

Systemic sclerosis (SSc) is a chronic autoimmune connective tissue disease characterized by immune dysregulation, widespread vasculopathy, and progressive fibrosis of the skin and various internal organs [Fig. 1] [1,2]. It is among the most severe rheumatic diseases, with the highest disease-related mortality [3]. Interstitial lung disease (ILD) and pulmonary arterial hypertension (PAH) are two major complications that significantly contribute to poor prognosis in patients with SSc [3]. While early diagnosis and therapeutic intervention can help slow disease progression, predicting individual disease trajectories remains difficult [1]. In addition to internal organ involvement, cutaneous symptoms—such as Raynaud's phenomenon, skin thickening, and digital ulcers—are common and significantly impair quality of life [4].

The pathogenesis of SSc is complex and multifactorial, involving a sustained interplay between aberrant immune activation, endothelial dysfunction, dysregulated vascular remodeling, and extracellular matrix (ECM) accumulation [1,2]. Recent studies have also highlighted the contribution of cellular senescence in SSc, particularly through the senescence-associated secretory phenotype (SASP), which perpetuates chronic inflammation and fibrosis [5].

Among the mediators implicated in these processes, plasminogen activator inhibitor-1 (PAI-1) has emerged as a molecule of considerable interest. PAI-1 is the primary physiological inhibitor of tissue-type and urokinase-type plasminogen activators (tPA and uPA) and is a key regulator of the fibrinolytic system [6]. It belongs to the serpin (serine protease inhibitor) family and is expressed by a variety of cell types, including endothelial cells (ECs), fibroblasts, macrophages, and alveolar epithelial cells [[6], [7], [8]]. Its expression is induced by multiple stimuli, including inflammatory cytokines, hypoxia, oxidative stress, and transforming growth factor-β (TGF-β) [6,7].

Beyond its classical role in suppressing fibrinolysis and stabilizing ECM deposition, PAI-1 plays broader roles in regulating angiogenesis, vascular remodeling, tissue repair, and cellular senescence [6,[9], [10], [11]]. These pleiotropic functions are highly relevant to the pathophysiology of SSc. For instance, increased PAI-1 expression has been observed in the skin, lungs, and serum of patients with SSc [8,[12], [13], [14]]. In the vasculature, PAI-1 contributes to endothelial dysfunction by impairing fibrinolytic balance, inhibiting angiogenesis, and promoting vascular rarefaction [11,15,16]. In the context of fibrosis, PAI-1 inhibits ECM degradation and facilitates fibroblast activation, thereby contributing to tissue stiffening and scarring [6,7,17]. Furthermore, recent findings suggest that PAI-1 is intricately involved in cellular senescence pathways, both as a biomarker and as an active mediator that reinforces the SASP and sustains the fibrotic and inflammatory milieu [18,19].

Given its multifaceted roles across several hallmarks of SSc pathogenesis—including vasculopathy, immune dysregulation, fibrosis, and senescence—PAI-1 represents a critical link between these processes. In this review, we summarize current knowledge on the function of PAI-1 in SSc, with a particular focus on its roles in vascular pathology, fibrotic remodeling, and senescence-associated tissue damage. A better understanding of these mechanisms may pave the way for novel therapeutic approaches targeting PAI-1 signaling in SSc.

## 2 Physiological function of PAI-1

PAI-1 is the principal physiological inhibitor of tPA and uPA, both of which convert plasminogen into its active form, plasmin [6,20]. Plasmin plays key roles in fibrin degradation and ECM remodeling: it not only dissolves fibrin clots but also activates multiple matrix metalloproteinases (MMPs), including MMP-1, MMP-3, MMP-9, and others [16,21,22]. Through these activities, plasmin contributes to ECM turnover and tissue repair. PAI-1, by suppressing the tPA/uPA-plasmin axis, acts as a brake on these processes, thereby limiting fibrinolysis and ECM degradation [20]. This antifibrinolytic property contributes to pathological fibrosis in several organ systems. Indeed, PAI-1 knockdown or knockout in experimental models enhances MMP activity, promotes ECM breakdown, and increases apoptotic sensitivity in fibroblasts—a phenomenon closely linked to cellular senescence, as described below [19,23,24].

PAI-1 also exerts complex and context-dependent effects on angiogenesis [25]. At low concentrations, PAI-1 forms a complex with vitronectin (VN), which stabilizes PAI-1 and subsequently promotes angiogenesis and facilitates endothelial cell (EC) migration [9]. For example, in models of oxygen-induced retinopathy and *in vitro* assays with human ECs, PAI-1 enhances vessel formation in response to angiogenic stimuli such as bFGF, PDGF-BB, and HGF [7,25]. Conversely, at higher concentrations, PAI-1 can exert angiostatic effects by forming inhibitory complexes with uPA. These complexes bind to the urokinase-type plasminogen activator receptor (uPAR) and associated molecules, including low-density lipoprotein receptor-related protein 1, α/β integrins, and VEGF receptor 2 [26,27]. Endocytosis of this multi-protein complex leads to signal termination and downregulation of angiogenesis.

In the vascular system, PAI-1 modulates vascular smooth muscle cell function and promotes neointimal formation [10]. Excessive PAI-1 expression can drive pathological vascular remodeling, narrowing the lumen and inducing tissue hypoxia [11,28]. Hypoxic conditions further enhance PAI-1 expression and SASP signaling, creating a vicious cycle of vascular activation [7,29]. Chronic hypoxia and PAI-1 upregulation have also been implicated in the induction of endothelial-to-mesenchymal transition (EndoMT), a process linked to fibrosis and vascular dysfunction [30,31].

In addition, PAI-1 plays a central role in regulating cellular senescence. PAI-1 overexpression in mouse embryonic fibroblasts (MEFs) induces proliferative arrest and cellular senescence via TGF-β stimulation [32,33]. In contrast, PAI-1 knockdown on endothelial cells sustains activation of the PI(3)K–PKB–GSK3β pathway, acquiring resistance to apoptosis and significantly extending cellular lifespan [32,34]. Under TGF-β stimulation, alveolar type II epithelial (ATII) cells upregulate both PAI-1 and p16, exhibiting hallmarks of senescence [19]. Senescent cells secrete proinflammatory and profibrotic factors, contributing to age-related tissue dysfunction. In the kidney, age-associated upregulation of PAI-1 in ECs promotes podocyte loss and glomerulosclerosis [35]. Notably, inhibition or genetic ablation of PAI-1 extends lifespan and alleviates age-related phenotypes in murine models and *in vitro* assays using human cells [[35], [36], [37]].

PAI-1 is also implicated in inflammatory responses. Elevated levels have been observed in conditions such as inflammatory bowel disease (IBD), obesity-related nephropathy, and severe COVID-19 infections [[38], [39], [40]]. In models of acute kidney injury, IL-6-induced PAI-1 upregulation contributes to inflammaging through the induction of the cell cycle inhibitor p21 [41].

Taken together, these findings illustrate that PAI-1 is a multifunctional protein with roles extending beyond fibrinolysis inhibition and ECM remodeling. It also regulates angiogenesis, vascular homeostasis, inflammation, and cellular aging. Through these diverse biological functions, PAI-1 serves as a key mediator at the intersection of hemostasis, tissue repair, and immune regulation. When dysregulated, however, PAI-1 can tip the physiological balance toward pathological outcomes, contributing to disease processes rather than resolution. The following section will examine how these mechanisms converge in SSc, where aberrant PAI-1 expression is implicated in vasculopathy, fibrosis, immune dysregulation, and cellular senescence.

## 3 Pathogenesis of SSc and association of PAI-1

### 3.1 Environmental and genetic factors contributing to the onset of SSc

SSc is a multifactorial disease involving both environmental and genetic contributors. Among environmental triggers, occupational exposures such as silica and organic solvents, as well as non-infectious agents like certain drugs, pesticides, silicones, and heavy metals, have been implicated in disease onset [42]. Although these factors are diverse, their pathogenic consequences often converge, leading to immune dysregulation of lymphocytes and macrophages. This immune imbalance promotes the secretion of cytokines and autoantibodies, which initiate vasculopathy and fibrosis [43].

Genetically, SSc shows familial clustering, with a higher incidence among first-degree relatives, suggesting a heritable component in disease susceptibility [44]. However, genetic predisposition alone appears insufficient to trigger overt disease, indicating a necessary interplay with environmental stimuli [42]. Genome-wide association studies (GWAS) have identified numerous risk loci, mostly in non-coding regions, that regulate gene expression [45]. These polymorphisms predominantly affect immune-related pathways—such as type I interferon signaling, T and B cell activation, and NF-κB signaling—but also implicate fibroblasts and ECs, linking immunity to the fibrotic and vascular manifestations of SSc [42].

### 3.2 Autoimmune responses and early disease development

Environmental triggers and genetic susceptibility together are thought to initiate autoimmune activation in SSc [42,46]. Notably, SSc-specific autoantibodies can be detected prior to the appearance of initial symptoms like Raynaud's phenomenon, puffy fingers, and morning stiffness [47]. Capillaroscopic findings in early-stage patients often show nailfold capillary abnormalities, indicating that microvascular structures are among the first targets of autoimmune attack [48].

### 3.3 Vasculopathy in SSc and its link to PAI-1

EC injury is a hallmark of SSc and plays a pivotal role in its pathogenesis [49,50]. Multiple factors—ranging from ischemia-reperfusion injuries associated with Raynaud's phenomenon to viral infections, oxidative stress, and autoantibodies—can trigger EC dysfunction [47,[51], [52], [53]]. Among autoantibodies, anti-EC antibodies (AECAs) are found in a large proportion of SSc patients and can induce apoptosis of dermal microvascular ECs *in vitro* [[53], [54], [55]]. Furthermore, AECAs promote the expression of adhesion molecules such as VCAM-1, ICAM-1, and E-selectin, facilitating immune cell infiltration and amplifying vascular inflammation [47,55]. These upregulated adhesion molecules promote immune cell infiltration into the stroma, including Th2/Th17 cells and macrophages [2,56,57]. These immune cells secrete inflammatory cytokines and pro-fibrotic factors, exacerbating vasculopathy [47,58].

Under physiological conditions, vascular repair is achieved through two complementary processes: angiogenesis and vasculogenesis, both of which are crucial for restoring vascular integrity after injury [59]. Vasculogenesis involves the recruitment and differentiation of bone marrow-derived EPCs, contributing to the formation of new blood vessels *de novo* [52]. In contrast, angiogenesis refers to the sprouting of new capillaries from pre-existing vessels, driven by the proliferation and migration of mature ECs at sites of vascular injury [60]. However, these processes are severely impaired in SSc. EPC numbers and functionality are reduced,and aberrant expression of both pro-angiogenic (e.g., VEGF) and angiostatic (e.g., endostatin) factors leads to ineffective neovascularization [52,61,62]. Capillary loss and capillary rarefaction, commonly observed in nailfold capillaroscopy, reflect this defective vascular regeneration [63].

PAI-1 has been implicated in this dysregulated vascular environment. Elevated serum levels of uPA, tPA, and PAI-1 have been reported in SSc patients compared to healthy controls [13]. PAI-1 is also upregulated in dermal ECs and fibroblasts of affected skin [14,17]. Mechanistically, PAI-1 inhibits plasminogen activation by binding uPA, thereby suppressing VEGF-mediated signaling and angiogenesis through the VEGF receptor 2 pathway [26,27]. Nonetheless, despite PAI-1's anti-angiogenic potential, VEGF and VEGFR levels remain paradoxically high in SSc tissue and serum [13,17,61,64]. These observations suggest that while PAI-1 may play a modulatory role, it is unlikely to be the central determinant of angiogenic failure in SSc. Instead, alternative factors—such as the angiostatic VEGF_165_b isoform—may more directly impair angiogenesis [65]. Persistent vascular rarefaction leads to chronic tissue hypoxia, further fueling the production of VEGF, TGF-β, and endothelin-1 (ET-1) [47,58,66,67]. These mediators promote EC activation and drive EndoMT, a key process wherein ECs acquire mesenchymal characteristics and contribute to myofibroblast accumulation [66,67]. PAI-1 is induced during EndoMT by several stimuli, including ET-1, TNF-α, TGF-β, and Wnt signaling [19,68,69]. However, its precise role in EndoMT remains ambiguous. For instance, aged PAI-1 knockout mice exhibit exacerbated cardiac fibrosis accompanied by elevated expression of EndoMT-associated markers, including MMP-2 and MMP-9, as well as TGF-β, suggesting enhanced EndoMT activity in the absence of PAI-1 [70]. Conversely, in another model, PAI-1 overexpression combined with bleomycin administration similarly promotes lung fibrosis, along with increased MMP-2 and MMP-9 expression [71]. These seemingly contradictory findings suggest that PAI-1 may exert context-dependent effects on EndoMT, potentially acting as either a facilitator or modulator depending on the tissue environment and pathological conditions. In the following sections, we will explore in more detail how dysregulation of the coagulation/fibrinolysis system, fibroblast activation, and cellular senescence—each influenced by PAI-1—contribute to the progression of SSc.

### 3.4 Impaired coagulation and fibrinolysis system

The coagulation and fibrinolysis systems are not only essential for hemostasis but also for the regulation of vasculogenesis and angiogenesis [22]. Dysregulation of these systems can aggravate vascular injury and endothelial cell apoptosis, leading to the development of vasculopathy and subsequent tissue fibrosis [72]. In patients with SSc, elevated levels of fibrinogen, von Willebrand factor, tPA, and PAI-1 have been observed, indicating a hypercoagulable state [13,53,73]. Furthermore, prolonged clot lysis time is commonly seen in patients with recurrent digital ulcers, and biopsy specimens from leg ulcers often reveal fibrin occlusive vasculopathy [74,75]. Tissue factor expression and fibrin deposition have also been reported in lung tissue from patients with SSc-ILD, supporting the notion of an intrinsic procoagulant tendency [76].

Among these coagulation abnormalities, PAI-1 is thought to play a particularly important role. By inhibiting fibrinolysis, PAI-1 may contribute to persistent fibrin accumulation, impaired vessel remodeling, and chronic inflammation. This prothrombotic milieu may exacerbate vascular dysfunction and fibrosis, particularly in small vessels. Therefore, dysregulated coagulation and fibrinolysis, with PAI-1 as a key mediator, are intricately involved in the vasculopathic component of SSc.

### 3.5 Tissue fibrosis through fibroblast activation

Tissue fibrosis in SSc results from sustained activation and differentiation of fibroblasts into myofibroblasts, which excessively produce ECM components such as collagen [42,77]. These myofibroblasts arise from multiple sources, including resident fibroblasts, bone marrow-derived fibrocytes, and ECs undergoing endothelial-to-mesenchymal transition (EndoMT) [2,78]. Various profibrotic factors—including IL-1β, IL-6, TGF-β, and TNF-α—contribute to this process, promoting fibroblast activation and EndoMT [15,58,79]. Fibrocytes, which are increased in the peripheral blood of SSc patients, also produce fibrotic and proangiogenic cytokines and can differentiate into myofibroblasts, further amplifying the fibrotic response [78].

Macrophages also play a crucial role in SSc fibrosis. M2-polarized macrophages, which are enriched in the skin and lungs of SSc patients, secrete a range of profibrotic mediators such as TGF-β and IL-10 [80]. Moreover, recent studies have identified diverse macrophage phenotypes, including hybrid subtypes exhibiting both M1-and M2-like characteristics [81]. Such hybrid macrophages have also been identified in SSc tissues, and their distribution appears to be influenced by immunosuppressive therapies [82]. Given this complexity, targeting M2 macrophages alone may be insufficient for effective antifibrotic therapy. Notably, several *in vitro* and *in vivo* studies suggest that PAI-1 can modulate macrophage polarization in a context-dependent manner [40,83]. Further research is needed to elucidate how PAI-1 influences the dynamics of macrophage subtypes in SSc-related fibrosis.

PAI-1 is increasingly recognized as a significant modulator of fibrotic processes. Its expression is elevated both systemically and locally in SSc-affected tissues [12,13,17,71]. In pulmonary fibrosis models, overexpression of PAI-1 in conjunction with intratracheal bleomycin (BLM) administration induces excessive collagen accumulation, particularly in the lungs, highlighting its pro-fibrotic potential [71,84]. Additionally, TGF-β stimulation of ATII cells upregulates PAI-1 expression along with key pro-fibrotic cytokines such as IL-4, IL-6, and IL-13, while hypoxia enhances PAI-1 production in macrophages [7,19]. These findings suggest that PAI-1 acts both as a downstream effector and upstream amplifier of pro-fibrotic signaling in the lung microenvironment. PAI-1 is also implicated in the pathogenesis of PAH, a major vascular complication of SSc characterized by vasoconstriction, thrombosis, and inflammation, ultimately leading to proliferative vasculopathy and fibrosis in small pulmonary arteries [85,86]. A previous study demonstrated that inhibition of GATA-6 in human pulmonary artery endothelial cells (PAECs) increases the expression of PAI-1 and ET-1, while decreasing VE-cadherin expression [85]. Notably, reduced GATA-6 expression has also been observed in SSc-derived PAECs [85], suggesting that downregulation of GATA-6 may contribute to PAI-1 upregulation and endothelial dysfunction in SSc-associated PAH.

In cutaneous fibrosis, PAI-1 expression is induced by thymic stromal lymphopoietin (TSLP), a cytokine secreted by CD163^+^ macrophages [87]. Studies using TSLP knockout mice have shown reduced PAI-1 expression in response to TGF-β stimulation, suggesting that both TSLP and TGF-β act as key regulators of PAI-1 expression and fibrotic responses in the skin [19,87]. PAI-1, induced by TGF-β stimulation, acts as a key mediator of mast cell infiltration into lesional skin and concurrently upregulates ICAM-1 expression on dermal fibroblasts, thereby facilitating mast cell adhesion [33,88]. Functional experiments in human dermal fibroblasts further demonstrate that PAI-1 knockdown reduces the expression of α-SMA and COL1A1 and impairs fibroblast migration [12]. These mechanisms contribute to skin fibrosis in both scleroderma mouse models and the skin of patients with SSc [17,84]. However, the contribution of PAI-1 to skin fibrosis remains somewhat controversial. While pharmacologic inhibition of PAI-1 in bleomycin-induced scleroderma models significantly reduces dermal thickness [12], other studies using PAI-1 knockout mice have reported no significant changes in dermal fibrosis following subcutaneous bleomycin administration [89], highlighting variability across experimental models. Thus, while PAI-1 appears to play a consistent role in pulmonary fibrosis, its contribution to cutaneous fibrosis may vary depending on disease context and model system.

Taken together, PAI-1 influences multiple cell types and signaling pathways involved in SSc fibrosis, including fibroblasts, fibrocytes, macrophages, and ECs. However, its precise contribution to fibrotic remodeling in various tissues requires further elucidation.

### 3.6 Senescent cell accumulation and disease progression

Cellular senescence, characterized by irreversible cell cycle arrest and acquisition of a proinflammatory secretory profile, is increasingly implicated in the pathogenesis and progression of SSc. Senescence can be triggered by environmental stress, oxidative damage, and chronic inflammation, leading to the accumulation of senescent cells in various tissues [90,91]. This contributes to immunosenescence, autoimmunity, and tissue dysfunction—hallmarks of chronic diseases including SSc [91,92].

Epidemiological data show that the mean age at SSc diagnosis is between 33.5 and 59.8 years, with early-onset cases being rare [43]. Moreover, older age at onset is associated with more severe disease manifestations, such as SSc-ILD and SSc-PAH, and with poorer overall prognosis [93]. Given that SASP secretion is elevated in senescent cells, these findings suggest that older patients may be more prone to inflammatory responses, thereby contributing to disease progression. Therefore, aging and senescence are key contributors to the pathogenesis and prognosis of SSc.

Molecular studies of SSc lungs reveal increased expression of senescence-related genes and activation of the p53 pathway, while telomere shortening is evident in ATII cells [94]. In the skin, endothelial cells, and immune cells of SSc patients, senescent cells accumulate progressively, correlating with disease duration and severity [92,94].

Senescent ECs exhibit characteristic morphological changes, including enlargement, misalignment, and strong adhesion to the basement membrane, which disrupts their alignment with blood flow [95]. These cells show reduced nitric oxide production due to eNOS downregulation, impairing vasodilation, and they also express elevated levels of adhesion molecules such as VCAM-1 and ICAM-1 [47,61]. In addition, senescent ECs secrete PAI-1, MMPs, and SASP components, all of which contribute to vasculopathy and promote EndoMT [96]. Furthermore, PAI-1 is known as a direct inhibitor of eNOS, reducing nitric oxide production, leading to aberrant vasoconstriction [97]. This abnormal response promotes local ischemia, ultimately leading to tissue fibrosis in SSc [47].

Similarly, senescent fibroblasts—abundant in the SSc dermis—produce chemokines such as CXCL10 and CCL2, as well as ECM components including COL1A1 and PAI-1 [98]. These factors contribute to chronic inflammation and tissue fibrosis, underscoring the close association of senescent fibroblasts with both inflammatory responses and fibrotic remodeling [5,6]. The accumulation of senescent cells across multiple SSc-affected tissues, along with their ability to secrete PAI-1 and other SASP factors, suggests that cellular senescence is a key driver of persistent inflammation and fibrosis in SSc. Given the growing interest in senescence-targeted therapies, further exploration of the relationship between senescent cells and PAI-1 may offer novel avenues for disease modification.

## 4 Potential impact of PAI-1 inhibition on the pathophysiology of SSc

Given the multifaceted roles of PAI-1 in SSc pathogenesis—including its involvement in fibrosis, vasculopathy, coagulation abnormalities, and cellular senescence—targeting PAI-1 represents a compelling therapeutic strategy [16,17,61,99]. PAI-1 is upregulated in the serum and lesional tissues of SSc patients and has been implicated in perpetuating a hypercoagulable state, promoting fibroblast activation, and impairing EC function. Its profibrotic effects are mediated by inhibition of fibrinolysis, promotion of myofibroblast differentiation, and induction of ECM accumulation. Furthermore, PAI-1 contributes to vascular remodeling by suppressing EC migration and tube formation and promoting EndoMT.

Supporting the pathogenic role of PAI-1 in SSc, urokinase-type plasminogen activator receptor (uPAR) knockout mice—which lack the ability to activate plasminogen via uPA—develop key features of SSc, including peripheral vasculopathy, dermal fibrosis, and interstitial lung disease [100]. These mice are increasingly recognized as an experimental model of SSc. Given that uPAR deficiency leads to impaired fibrinolysis and compensatory upregulation of PAI-1, these findings further implicate PAI-1 as a central mediator of disease and strengthen the rationale for its therapeutic inhibition.

In SSc, where vascular dysfunction likely precedes and initiates the fibrotic cascade, PAI-1 inhibition may help restore endothelial homeostasis. By attenuating the procoagulant milieu—characterized by elevated fibrinogen, von Willebrand factor, and reduced fibrinolytic activity—PAI-1 blockade could ameliorate digital ulcers and reduce the risk of organ ischemia. In addition, by lifting the suppression of plasmin activity, PAI-1 inhibition may enhance ECM turnover and facilitate the resolution of established fibrosis.

Importantly, PAI-1 also plays a crucial role in cellular senescence, particularly in vascular and mesenchymal cells. Senescent ECs and fibroblasts accumulate in SSc and contribute to chronic inflammation through the secretion of SASP factors. PAI-1 is not only a marker of senescence but also actively reinforces the senescent phenotype via the p53/p21 pathway. *In vitro* studies have shown that PAI-1 inhibition can reverse senescence-associated changes in human umbilical vein endothelial cells (HUVECs) and promote apoptosis of senescent ECs via the Fas/FasL pathway [54,101]. This suggests that targeting PAI-1 may allow for selective clearance of deleterious senescent cells, thereby reducing vascular inflammation and dysfunction [102]. Although the induction of apoptosis might raise concerns in the context of SSc, it could also facilitate the removal of chronically damaged cells and improve tissue homeostasis [92,103].

From an immunological standpoint, PAI-1 may influence macrophage polarization. While M2 macrophages are traditionally associated with tissue repair and fibrosis, recent data indicate that PAI-1 modulates macrophage plasticity in a context-dependent manner. *In vivo* models have demonstrated that PAI-1 inhibition shifts macrophage phenotypes and decreases profibrotic cytokine production. This may be especially relevant in SSc, where hybrid macrophages expressing both M1 and M2 markers are abundant and contribute to persistent tissue injury.

Pharmacological inhibition of PAI-1 has recently been explored in human clinical settings. TM5614, an orally bioavailable PAI-1 inhibitor, has been investigated in investigator-initiated clinical trials targeting chronic myeloid leukemia (CML), malignant melanoma, and COVID-19–associated pneumonia [[104], [105], [106]]. In CML, the addition of TM5614 enhanced molecular responses in patients with suboptimal responses to prior tyrosine kinase inhibitors [104]. In patients with unresectable melanoma refractory to anti–PD-1 antibody therapy, TM5614 combined with nivolumab achieved an overall response rate of approximately 26 % [105]. Although the COVID-19 study could not produce definitive efficacy data due to the subsiding pandemic, no serious adverse events were reported in any of the trials [106]. Although the therapeutic efficacy of TM5614 in SSc has not yet been reported, an *in vivo* study using Tiplaxtinin, another PAI-1 inhibitor, showed significant suppression of skin fibrosis in a mouse model [12]. These findings suggest that PAI-1 inhibition holds promise as a therapeutic approach for SSc.

Despite the promising preliminary data, PAI-1 inhibition is not without potential risks. Given the physiological role of PAI-1 in hemostasis, excessive suppression may increase the likelihood of bleeding complications such as ecchymoses, epistaxis, or hematoma formation [107]. Therefore, thorough safety assessments are required, especially in SSc patients with inherent vascular fragility. Careful optimization of dosage, treatment timing, and patient selection will be essential to achieve a favorable risk-benefit profile.

In summary, PAI-1 inhibition offers a multifaceted approach to SSc treatment by targeting fibrosis, vasculopathy, cellular senescence, and immune dysregulation simultaneously. Unlike therapies aimed at individual cytokines or signaling pathways, PAI-1–targeted interventions have the potential to disrupt core mechanisms of disease progression across multiple organ systems. If proven effective and safe, PAI-1 inhibitors may significantly advance the treatment landscape for SSc, offering therapeutic benefit for its most severe manifestations, including cutaneous sclerosis, ILD, and PAH. Further preclinical and clinical studies are warranted to fully evaluate the therapeutic potential of this approach.

## 5 Conclusion

**Fig. 2:**
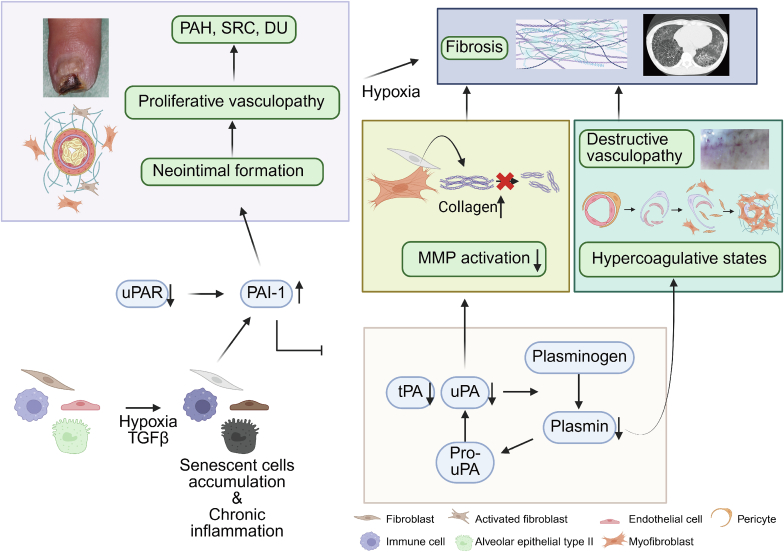
Role of PAI-1 in the pathogenesis of SSc. Various stressors, such as hypoxia and TGF-β, induce cellular senescence and upregulate PAI-1 expression in fibroblasts, endothelial cells, alveolar type II epithelial cells, and immune cells. PAI-1 suppresses the fibrinolytic system by inhibiting urokinase-type (uPA) and tissue-type plasminogen activators (tPA), thereby reducing plasmin generation. This leads to a hypercoagulable state, destructive vasculopathy (loss of small vessels), decreased matrix metalloproteinase (MMP) activation, and excessive collagen deposition. In parallel, PAI-1 promotes neointimal formation and proliferative vasculopathy (occlusion of arterioles and small arteries with fibro-proliferative change), which ultimately contributes to the development of pulmonary arterial hypertension (PAH), scleroderma renal crisis (SRC), and digital ulcers (DU). These processes converge to drive disease progression in SS.c.

SSc is a multifactorial disease characterized by immune dysregulation, vasculopathy, fibrosis, and cellular senescence. Among the molecular mediators involved, PAI-1 emerges as a key contributor to disease progression through its influence on coagulation, ECM remodeling, endothelial dysfunction, and senescent cell accumulation [Fig. 2]. Elevated PAI-1 levels in serum and tissues underscore its clinical relevance, and accumulating evidence points to its central role in SSc pathophysiology.

PAI-1 inhibition offers a promising therapeutic avenue by simultaneously addressing multiple pathological axes. It holds potential not only to reduce fibrosis and restore vascular function but also to modulate inflammation and immunosenescence. Although clinical validation is still required, targeting PAI-1 could represent a paradigm shift in SSc treatment, moving beyond symptom control toward disease modification.

## Declaration of generative AI and AI-assisted technologies in the writing process

During the preparation of this manuscript, the author utilized ChatGPT (OpenAI) to improve the clarity and language of the text. All content was subsequently reviewed and edited by the authors. The authors take full responsibility for the final version of the manuscript.

## Declaration of competing interest

There are no conflicts of interest to declare.
